# Jasmonates—the Master Regulator of Rice Development, Adaptation and Defense

**DOI:** 10.3390/plants8090339

**Published:** 2019-09-09

**Authors:** Hieu Trang Nguyen, Huong Thi Mai To, Michel Lebrun, Stephane Bellafiore, Antony Champion

**Affiliations:** 1 Institut de Recherche pour le Développement (IRD), Université de Montpellier, UMR DIADE, UMR IPME, UMR LSTM, 34394 Montpellier, France; 2 University of Science and Technology of Hanoi (USTH), Vietnam Academy of Science and Technology (VAST), LMI-RICE2, 18 Hoang Quoc Viet, Cau Giay district, 100000 Hanoi, Vietnam

**Keywords:** jasmonates, signaling, defense response, development, rice

## Abstract

Rice is one of the most important food crops worldwide, as well as the model plant in molecular studies on the cereals group. Many different biotic and abiotic agents often limit rice production and threaten food security. Understanding the molecular mechanism, by which the rice plant reacts and resists these constraints, is the key to improving rice production to meet the demand of an increasing population. The phytohormone jasmonic acid (JA) and related compounds, collectively called jasmonates, are key regulators in plant growth and development. They are also one of the central players in plant immunity against biotic attacks and adaptation to unfavorable environmental conditions. Here, we review the most recent knowledge about jasmonates signaling in the rice crop model. We highlight the functions of jasmonates signaling in many adaptive responses, and also in rice growth and development processes. We also draw special attention to different signaling modules that are controlled by jasmonates in rice.

## 1. Introduction

Jasmonic acid and its related derivatives are polyunsaturated fatty acid-derived phytohormones occurring ubiquitously in land plants. These lipid-based signal molecules are involved in several aspects of plant growth, adaptation to various environmental constraints, and in plant defense against biotic attacks [1,2,3,4,5,6,7,8]. A large portion of our knowledge in the JA biosynthesis pathway and signaling comes from the studies in the dicotyledonous plant model, such as Arabidopsis (*Arabidopsis thaliana*), and tobacco (*Nicotiana tabacum*). In recent years, several efforts have been made on exploring jasmonates mechanisms in crop species. Rice is among the most important crops worldwide, feeding more than half of the world’s population. Rice is also a model plant for molecular studies in monocotyledonous plants. Understanding the molecular mechanism of jasmonates signaling is expected to not just facilitate the breeding programs for rice, but also for other main cereals. In rice, all homologs of JA biosynthesis and signaling components have been identified, however, only a scant number of genes have been characterized for their functions in recent years (Table S1). Remarkably, the rice genome consists of a higher number of homologs encoding for the receptors and repressors, with three *CORONATINE INSENSITIVE* (*OsCOI*) genes (designated as *OsCOI1a*, *OsCOI1b*, and *OsCOI2*) [9] and fifteen *JASMONATE-ZIM-DOMAIN PROTEIN* (*OsJAZ*) genes (designated as *OsJAZ1* to *OsJAZ15*) [10], as compared to one *AtCOI* and thirteen *AtJAZ* genes in Arabidopsis. The presence of three closely related F-box protein receptors in rice suggests that there might be more possibilities of distinguishing different forms of jasmonates other than JA-Ile at the perception level [11]. Indeed, a recent study in Arabidopsis suggested that, beside JA-Ile as the main endogenous bioactive compound, the AtCOI1 protein could also perceive 12OH-JA-Ile as the ligand to initiate the signaling in response to wounding [12]. In addition, Monte et al. (2018) [13] identified the JA-Ile precursor dinor-OPDA as the ligand of *Marchantia* MpCOI1. The higher number of receptors and repressor proteins found in rice suggest a higher number of possible COI-JAZ interactions, which may lead to the fine-tuning of JA perception and signaling. Actually, it has been shown that each OsCOI homolog interacts with different subsets of OsJAZ proteins [9]. For example, OsJAZ1, OsJAZ4, and OsJAZ7 interact with OsCOI1b, but not OsCOI1a, whereas OsJAZ5 interacts with OsCOI2 and OsCOI1a but not OsCOI1b (the number of OsJAZ is referred to in [10]). In this review, we discuss the recent significant findings of jasmonates signaling in rice. 

## 2. The Roles of Jasmonates in Rice Growth and Development

Jasmonates are involved in rice growth and development, ranging from seed germination, seedling growth, root growth, photomorphogenesis, gravitropism, senescence, flower development, fertility, and seed development. Some of these aspects have been reviewed intensively, such as the role of jasmonates in photosynthesis [11,14], and the interaction between jasmonates and other hormonal signaling in regulating plant growth and development [15,16]. In this section, we will focus on the role of jasmonates in spikelet development and vegetative growth for this important food crop. 

### 2.1. Jasmonates Play a Determinant Role in Spikelet Development and Fertility in Rice

The most prominent and best-described role of jasmonates in plant development is its regulation of reproductive organs differentiation and fertility, which are, strictly, genetically controlled processes. The hallmarks observed in JA biosynthetic mutants are the defects in flower structure and sterility, as reported in different plant species, including Arabidopsis (*Arabidopsis thaliana*) [17,18,19,20,21], tomato (*Solanum lycopersicum*) [22,23,24], cotton (*Gossypium hirsutum*) [25], moss (*Physcomitrella patens*) [26], and maize (*Zea mays*) [27,28]. In rice plants, strikingly, alterations in spikelet structure were observed in all biosynthetic mutants identified so far, as well as in several signaling genes (Table S1). Some of those defective phenotypes in the spikelet organ include abnormal or reduced stamens, reiterative glume-like structures, stigma-like organs, and impaired anther dehiscence (Table S1). The number and the degree of symptoms are likely correlated with the defective level in the JA biosynthesis pathway. Among the biosynthetic mutants, *cpm2/hebiba* is impaired in the single copy gene *ALLENE OXIDE CYCLASE (OsAOC)* in the rice genome; therefore, these mutants accumulated almost no OPDA, JA, and other derivatives [29,30]. As a result, *cpm2/hebiba* plants showed all above described flower-related phenotype, and exhibited complete male sterility, while other biosynthetic mutants are partially sterile [29,30,31,32,33,34,35,36]. So far, the signal induces the accumulation of jasmonates during spikelet development and fertility remains elusive. Recently, a mutation in *OsPEX5* gene, which encodes a peroxisomal targeting sequence 1 (PTS1) receptor protein, also caused abnormal spikelet morphology by reducing JA biosynthesis [37]. *OsPEX5* regulates spikelet development via transporting the JA biosynthesis enzyme OsOPR7 into peroxisomes, where JA biosynthesis occurs. As a result, in the *ospex5* mutant, OsOPR7, diffused in the cytosol instead of being localized to peroxisomes, therefore disturbing JA biosynthesis [37]. Further analysis should be done to determine if the *ospex5* mutant phenotype is a result of low JA, or a result of JA precursors accumulating in the cytosol.

**Figure 1:**
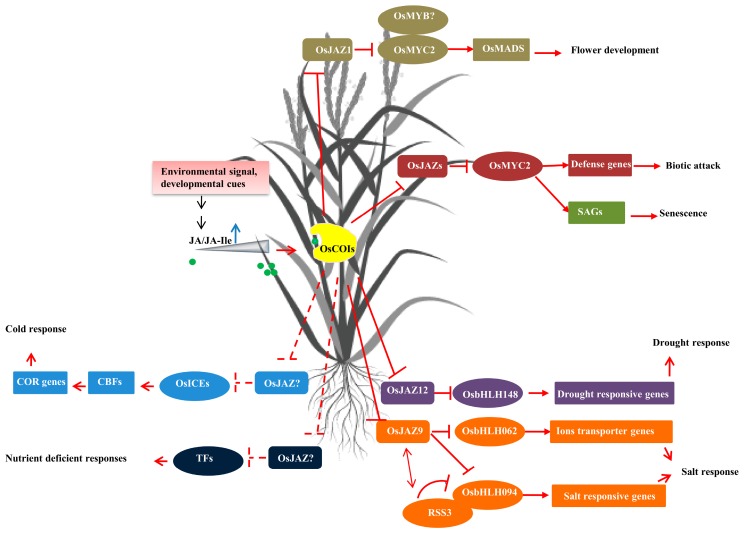
Summary of core jasmonates-regulated signaling modules involved in specific response in rice. The core signaling module constitutes JA-Ile/OsCOI/OsJAZ/TFs. Input signals from biotic attacks or abiotic stresses, such as salinity, cold, drought…, trigger the accumulation of JA/JA-Ile. JA-Ile is perceived by the OsCOI protein receptor and promotes degradation of JAZ proteins through the 26S proteasome manner. Degradation of OsJAZ relieves repression on the OsJAZ-interacting transcription factors (such as OsMYC2, OsbHLH062, and OsbHLH094) that govern specific physiological output responses involved in growth, development, and tolerance to biotic and abiotic stresses. However, this simplified scheme does not negate the fact that many OsJAZ proteins functionally interact with multiple transcription factors. Arrows represent positive regulatory actions. Lines ending in a flat head indicate a negative regulatory action. Dashed lines represent interactions that have not been experimentally confirmed. Double-headed arrows indicate that two proteins interact.

The regulation role of jasmonates in rice spikelet development and fertility relies on the core, conserved signaling module, which constitute JA-Ile/COI/JAZ/TFs. In this conserved model, the endogenous concentration of JA-Ile in the inflorescence needs to surpass the threshold level to trigger degradation of JAZ repressor that allows induction of TFs-mediated transcription of flower-related genes. Rice lines with reduced level of JA-Ile by mutation of the *JASMONYL-L-ISOLEUCINE SYNTHASES 1* (*OSJAR1,* which functions in conjugating JA and isoleucine) gene, or overexpression of the *JASMONIC ACID CARBOXYL METHYL TRANSFERASE* (*AtJMT,* which methylates JA into MeJA) gene also showed altered spikelet structure and reduced fertility [36,38]. Among the three *OsCOI*, *OsCOI1a,* and *OsCOI1b*, but not *OsCOI2,* were able to restore the male sterility of *coi1* mutant in Arabidopsis [9]. Knockout or knockdown of *OsCOI1a* or *OsCOI1b* did not result in any abnormality in the flower phenotype, suggesting that *OsCOI* genes might have a distinct function other than flower development [39,40]. Similarly, the JAZ protein family, which is far much better studied in Arabidopsis, also showed highly redundant activity with no obvious flower-related phenotype or male sterility reported after a knockout of a single *JAZ* gene in Arabidopsis [41]. Interestingly, in rice, a mutation in the *OsTIFY3*/*OsJAZ1* gene (LOC_Os04g55920) was identified with extra glume-like structures between the sterile lemma and lemma and altered floral organ numbers and identities, hence named as *extra-glume 2 (eg2-1D*) [31] (Table S1). The Ala-to-Gly mutation in the Jas domain of OsJAZ1 in *eg2-1D* blocked the eg2-1D-OsCOI1b interaction, therefore prevented it from ubiquitination and degradation. It was shown that, in *eg2*-*1D* mutant, the expression of several floral organ identity genes that belong to E-class genes (*OsMADS1*, *OsMADS7*, and *OsMADS8*), that play a key role in specifying inflorescence and spikelet development in rice, was repressed. Furthermore, it was found that OsMYC2 can directly bind to the promoter and regulate the expression of *OsMADS1*, *OsMADS7*, and *OsMADS14*, suggesting that OsMYC2 is the transcription factor involved in flower development in rice [31,37]. Thus, these results revealed the JA-mediated signaling module in regulating rice spikelet development, which is brought about by JA-Ile/OsCOIs/OsJAZ1/OsMYC2/OsMADSs (Figure 1) [31]. Several R2R3-MYB TFs, such as MYB21 and MYB24, that act together with MYC2, MYC3, MYC4, and MYC5 by forming MYB-MYC complexes to control stamen development, were identified in Arabidopsis [7,42,43]; however, the function of *OsMYB* genes involved in stamen development has not been reported in rice. Along with the *eg2-1D* mutant, overexpression of the dominant-negative JAZ factors with mutated jas domains (namely *mJAZ3ox, mJAZ6ox, mJAZ7ox,* and *mJAZ11ox*) caused pleiotropic defects in rice spikelet development with similar phenotypes observed in the *eg2/osjaz1* mutant [44] (Table S1). The aberrant morphogenesis observed in different *mJAZox* lines could be explained by the dominance of the stabilized repressors over the endogenous OsJAZs, thus restraining the activation of OsMYC2 and downstream *OsMADS* genes. However, these defected phenotypes should not be attributed to the function of a single gene. Hence, the *OsJAZ1* gene, so far, remains the only member of the JAZ family identified that is involved in spikelet development in rice.

### 2.2. Jasmonates Negatively Regulate Vegetative Growth

Beside the conspicuous phenotype on the spikelet, mutations in JA biosynthesis pathway promote vegetative growth and development [29,32,33]. For example, in favorable conditions, the *cpm2/hebiba* and the *pre* mutants (Table S1) developed longer primary root length, long leaves (especially from leaf two to leaf four), underwent the transition from juvenile phase to adult phase sooner, than the wild type plants by about 5–7 days [29,33]. These observations suggested that JA deficient plants grow better in non-stress conditions. However, these mutants also show a hypersensitivity to pathogen attacks (see details in the following section). Moreover, transgenic rice plants that overexpressed JA repressors, such as *OsJAZs* or *OsNINJA1* genes, were less sensitive to JA-induced root inhibition, showed better root growth and gained higher yield in non-stress conditions [45,46,47]. To the contrary, transgenic rice plant overexpressing the *OsMYC2* gene bypassed the repression by OsJAZs to constitutively induced JA-responsive genes and showed a dwarf phenotype under normal growth conditions [48]. Consistently, exogenous application of JA inhibited various aspects of plant growth, including primary root growth, root biomass, and shoot biomass [49]. Collectively, these pieces of evidence demonstrated that jasmonates signaling negatively regulates vegetative growth. 

The plant growth–defense balance is regulated mainly by the crosstalk gibberellic acid (GA)-jasmonic acid, in which GA prioritizes growth and JA prioritizes defense [2]. The GA-JA crosstalk is mediated through the physical interaction of the DELLAs-JAZs protein, which functions as a repressor of GA, JA signaling, respectively. During the JAs signaling, JAZs proteins are degraded to release the DELLA protein, which in turn interact with PIF3 (the master transcription factor in the GA signaling pathway) and suppress GA-mediated plant growth [2]. In rice, the suppression of *OsCOI1*a and *OsCOI1b* genes exhibited similar phenotypes to rice plants overproducing GA. MeJA treatment facilitates the rice DELLA protein’s SLR1 accumulation and delays GA-regulated SLR1 degradation in wild-type rice plants [40]. The JA-induced root elongation inhibition is also mediated through antagonism with the auxin signaling pathway, as demonstrated in Arabidopsis [50]. It was found that JA-induced flavonoids (e.g., anthocyanins) act as negative regulators of auxin transport [51]. For example, naringenin, an early precursor in the flavonoid biosynthetic pathway, inhibits primary root elongation as a result of its inhibitory effects on auxin transport [52]. In rice, naringenin is the precursor of the phytoalexin sakuranetin, which plays an important role in rice defense. Both naringenin and sakuranetin were strongly induced by exogenous treatment of JA, attacked by the rice blast fungus *Magnaporthe oryzae*, CuCl2 treatment, and UV irradiation; such conditions also resulted in primary root growth inhibition in rice [30,53,54,55].

## 3. Jasmonates Play a Central Role in Rice Immunity in Response to Various Biotic Attacks

It has been nearly 30 years since the role of jasmonates in regulating the biosynthesis of defensive proteinase inhibitors was discovered in tomato and tobacco leaves [56]. Since then, it has attracted a lot of research that has highlighted the important role of jasmonates in plant immunity against a broad spectrum of plant-associated organisms, ranging from microbial pathogens to vertebrate herbivores. Mainly from studies in Arabidopsis and tomato, it is generally accepted that the JA-triggered immunity (JATI) is responsible for the resistance to necrotrophic pathogens and insect herbivores, while SA-triggered immunity (SATI) is considered to be the main player in resistance against biotrophs [1,4]. However, this general acceptance should be reconsidered, regarding the exceeding number of biotrophic pests whose fitness is curtailed by JATI. In particular, in rice plants, JATI has been implicated in response to diverse pests and pathogens, most of them having a biotrophic feeding style, including pathogenic bacteria, (hemi) biotrophic fungi, nematodes, leaf/root chewing insects, piercing-sucking insects, virus, parasitic plants, vertebrate herbivores, fungus gnats, and some crustaceans (Table 1). 

**Figure 2:**
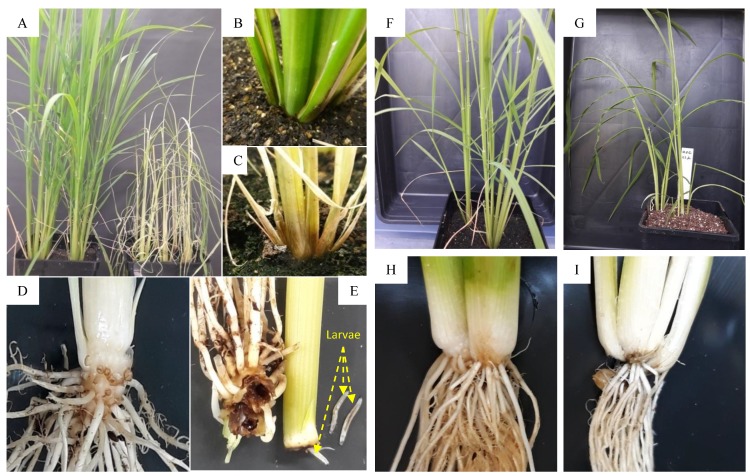
The JA biosynthetic *osaoc* mutant succumbs to attack by larval of the soil fly fungus gnats *Bradysia spp*. (Diptera: Sciaridae). In the greenhouse conditions, homogenous mutant plants (*osaoc/osaoc*) were planted next to the heterozygous and wild type plants in the soil. Larvae were able to attack to the stem of the homogenous mutants, result in wilting and plants dying after few days, while the wild type plants were just attacked on some roots and had no obvious symptoms on the above-ground part. The assay was repeated at least three times with the total observation of homozygous mutants of more than 100 plants. When a fly trap was applied, or the soil treated with larvacide (VectoBac), or 10 µM of JA applied through spraying on leaf or soil, the mutant plants were able to survive until flowering. The figure shows the wild type plants (**A** (plants on the left), **B**, and **D**) and the homozygous mutant plants (**A** (plants on the right), **C**, and **E**) with the wilting symptom on their leaves and the larvae found in the stem of attacked mutant plants. (**F**,**H**) Healthy heterozygous plants without insect attack. (**G**,**I**) Healthy *osaoc* mutant plants without insect attack.

Remarkably, the serendipitous discovery that some organisms which do not usually feed on living plants, such as the detritivorous crustacean *Porcellio scaber* and *Armadillidium vulgare,* suddenly become herbivores on jasmonate-deficient Arabidopsis and rice plants [82]. Similarly, observations in Arabidopsis *aos*, maize *opr7opr8*, and tomato *jai1* mutants showed that they suffered 100% mortality from root rot disease caused by the oomycete pathogen *Pythium irregulare* [1,83,84]. We also assayed that the rice *osaoc* mutant in rice Kitaake succumbed to attack by larvae of fungus gnats *Bradysia* spp., highliting the role of JAs in unusual interactions (Figure 2). The larval stage, which feeds mainly on decaying plant material, fungi, and algae [85], now, has become a dreadful enemy for the *osaoc* mutant plant. On wildtype and heterozygous plants, the larvae can attack only a few roots but do not result in any symptoms on the above-ground part of the plant. However, on the homozygous mutant plants, larvae can easily feed on the roots and tunnel into stems, thus interfering with the ability of plants to uptake and transport water and nutrients, which results in wilting and dying after few days. As expected, isolating the mutant from fungus gnats using a fly trap or treating the soil with the biological larvacide (commercially named Vectobac) or applying 10 µM JA to the plant was efficient to protect the mutant from attack by the fungus gnats larvae. Collectively, these reports highlight the importance of JATI for the survival of the plant under attack from numerous surrounding invaders in their environment.

The central role of jasmonates in induced immunity is grounded in four general observations. First, biotics attack result in the up-regulation of JA biosynthetic genes and a rapid accumulation of JA and JA-Ile, at both damaged sites and systemic tissues [30,57,62,64,75,86]. Second, induced accumulation of jasmonates activate the signaling cascade, leading to the production of pathogenesis-related (PR) proteins, anti-nutritional proteins, and secondary metabolites that have established roles in defense, such as phytoalexins [72,78,87]. Third, mutants or transgenic plants overexpressing components of jasmonates signaling show altered resistance to pathogens attack [30,47,48,57,59,65,67,68,71,75,81,88,89]. For example, the silencing of the *LIPOXYGENASE (OsHILOX)* or mutant of the *ALLENE OXIDE CYCLASE (OsAOC)* genes, which are involved in the JA biosynthetic pathway, reduces plant resistance to the rice striped stem borer (SSB, *Chilo suppressalis*), the rice root feeding insect (*Diabrotica balteat*), blast fungus (*Magnaporthe oryzae*), the root-knot nematode (*Meloidogyne graminicola*), and a parasitic plant (*Striga hermonthica*) [30,60,62,70,76]. Suppression of the *OsCOI1a* gene increases susceptibility to the rice leaf folder (*Cnaphalocrocis medinalis*) [63], and the *Rice black-streaked dwarf virus (RBSDV)* [63,75]. Overexpression of the group I GH3 family genes, which encoding JA-Ile synthetase, enhances tolerance to the blight disease caused by *Xanthomonas oryzae* pv. *oryzae (Xoo)* [59]. Finally, the jasmonates signaling pathway is the primary target modulated by many plant pathogens to hijack the plant defense response during host–pathogen interaction [6,73,77,90,91]. For example, *Magnaporthe oryzae,* which causes the blast disease in rice, secretes an antibiotic biosynthesis monooxygenase (Abm) that converts endogenous free JA into 12OH-JA to prevent the induction of JATI during and after the invasion to facilitate host colonization [77].

Recent genetic approaches and the exogenous application of synthetic hormone analogs have investigated the interplay between JA and other plant hormones during pathogen attacks. Among these phytohromones, strigolactones (SLs) have recently been presented as potential regulators since they can increase or decrease infection depending on the plants, tissues, and pathogens. In some pathosystems such as tomato/*Botrytis cinerea* or tomato/*Alternaria alternate*, SLs reduce infection [92], whereas in rice, *Meloidogyne graminicola* requires SLs to promote infection [93]. Interestingly, at the onset of infection *M. graminicola* induces the SLs biosynthesis which then suppresses the JA pathway in rice [93]. Therefore, SLs appear to be the primary target of some phytopathogens in order to establish a compatible interaction in a manner that involves JA.

Another strategy to hijack the host immunity through activating the *OsmiR319b* pathway, by targeting the *TEOSINTE BRANCHED/CYCLOIDEA/PROLIFERATING CELL FACTOR1 (OsTCP21)* transcription factor, was exploited by both *M. oryzae* and the *Rice ragged stunt virus (RRSV)* [74,86]. *OsTCP21* was found as a positive regulator of rice immune system, particularly the JATI [74]. An unknown factor from the virulence strain *M. oryzae* G11or *RRSV* induced expression of *OsmiR319b* resulting in the suppression of *OsTCP21* and preventing expressions of *OsTCP21* target genes, such as *OsLOX2* and *OsLOX5*. As a result, *M. oryzae* and *RRSV* could better colonize on JATI-weakened host plants [74,86].

The expression of the majority of genes involved in JATI is regulated by the core signaling module constituted by JA-Ile, COI1, JAZ, MYC2, and MYC2-like [50,90,91,94,95]. Perception of danger signals, such as microbe-associated molecular patterns (MAMPs), herbivores-associated molecular patterns (HAMPs), and damage-associated molecular patterns (DAMPs), by pattern recognition receptors (PRRs) at the cell surface activate intracellular signaling cascades, which involve MAP kinase pathways, calcium ion-sensing proteins, and reactive oxygen species (ROS), and raise the level of endogenous JA/JA-Ile [1]. In rice, the plasma membrane-localized LRR-RLK, OsLRR-RLK1, was identified as a potential receptor in the perception of herbivory-associated molecular patterns. OsLRR-RLK1 acts upstream of mitogen-activated protein kinase cascades, and positively regulates SSB-elicited levels of jasmonic acid, trypsin protease inhibitor activity, and plant resistance towards SSB [66]. Among the three *OsCOI* genes, the mutant of *OsCOI1b* revealed a higher susceptibility to infection by the *Rice black-streaked dwarf virus* [75]. The functional homolog of *MYC2* was also characterized in rice [48,53]. OsMYC2 interacted with all OsJAZ protein, except OsJAZ14, through its highly conserved JAZ-interacting domain (JID) [48]. Transgenic rice plants overexpressing *OsMYC2* exhibited a JA-hypersensitive phenotype and were more resistant to rice bacterial blight through constitutively up-regulation of several defense-related genes, including some PR genes [48]. To the contrary, in the *OsMYC2*-knockdown plants (*osmyc2RNAi*), JA-inducible expression of many defense-related genes, for example, chitinases and proteinase inhibitors, as well as of specialized metabolites, especially defensive compounds, was compromised [53]. Furthermore, a substantial change was noted in the expression of distinct types of transcription factors, such as *MYB*-type factors, likely depicting the importance of *OsMYC2* in the rice defense responses [53]. Recently, the role of *OsNINJA1* gene in rice jasmonates signaling was reported [47]. Similar to its homolog in Arabidopsis, OsNINJA1 is able to interact with several OsJAZ proteins (except OsJAZ14 and OsJAZ15) in a C domain-dependent manner. *OsNINJA1*-overexpressing rice plants diminished the expression of *OsMYC2*-responsive pathogenesis-related (PR) genes and senescence-associated genes, and were more susceptible to *Xoo* [47]. The highly redundant activity in the *JAZ* family makes it difficult to identify, if there is one, the member of this family involved in specific response to a particular pathogen. For example, loss-of-function of any single gene among the thirteen *JAZ* genes in Arabidopsis did not result in any significant change in plant susceptibility and growth [96]. It was reported that constitutive activation of anti-insect defense and inhibition of vegetative growth was only observed in the higher-order mutants, such as the *jaz* quintuple (*jaz*Q), or the *jaz* decuple (*jaz*D) [96,97]. Just a few mutants in the *OsJAZ* genes have been isolated so far (Table S1) and none of them was reported to affect rice resistance. Although, some studies using transgenic rice plants overexpressing *OsJAZ8ΔC*, which lacks the Jas domain, reported that the *OsJAZ8ΔC* plant was insensitive to JA and negatively regulated JA-induced resistance to *Xoo* in rice [57]. However, dominantly expressed stabilized OsJAZ8ΔC can inhibit the global jasmonates signaling, therefore, any defected phenotype cannot be attributed to the function of *OsJAZ8*.

## 4. The Role of Jasmonates in Response to Abiotic Stress in Rice

Abiotic agents, such as salinity, drought, extreme temperature, and lack of essential nutrient components in the soil greatly reduce rice yield. The involvement of jasmonates signaling in different abiotic stress responses in rice has been implicated in several studies. Commonly, the conclusions came from the experiment with exogenous application of jasmonates and/or using mutants/transgenic plants that have altered in JA signaling. However, the role of jasmonates signaling in some responses (e.g., to salt and drought stress) remains controversial. Evidence for both positive and negative role of jasmonates in these responses has been raised. In this section, we review the major findings that indicate the involvement of jasmonates signaling in abiotic stress response in rice. We underline the fact that these studies have been performed in different conditions and with different genetic backgrounds that might be relevant to account for the different conclusions.

### 4.1. Roles of Jasmonates in Salt Stress

Rice plants are generally categorized as a typical glycophyte, which are sensitive to high sodium content in their environment, especially in their early seedling stages. Salinity has become the major abiotic stress limiting rice production worldwide, especially in the coastal areas. Salt stress mainly triggers three harmful effects to the plant: (i) Osmotic stress arises from NaCl-induced reduction of the soil solute potential, resulting in reduction of the hydraulic conductance, water and solute uptake by plants, (ii) specific ion toxicity stress arises from accumulation of noxious quantities of Na^+^ in the cells and tissues of the plant which lead to induction of cytosolic K^+^ efflux and consequently an imbalance in cellular homeostasis, (iii) oxidative stress by uncontrolled production of reactive oxygen species (ROS) as the consequence of water shortage, including superoxide radicals (O^-^_2_), hydrogen peroxide (H_2_O_2_), and hydroxyl radicals (OH^–^) [5,98,99].

For a long time, jasmonates has been reported for its involvement in response to salinity stress in rice. Firstly, it was shown that the endogenous MeJA level in rice root (*Oryza sativa* subsp. *indica* cv Taichung N1) increased concomitantly with the concentration of sodium in the medium [100]. Exogenous application of MeJA triggered the accumulation of several pathogen-related proteins and most JA-responsive proteins were also accumulated in rice roots when subjected to salt stress, suggesting that jasmonates may play a role in response to salt stress [100]. Indeed, *JIOsPR10*, a gene induced by both JA and salt stress, enhanced salt tolerance when overexpressed in rice [78]. In support of these results, Kang et al. (2005) [101] studied the effect of exogenous JA application in two salt-stressed rice cultivars, which showed contrasted performance under salt stress (*Oryza sativa* subsp. *japonica* cv Dongjinchalbyeo and Dongjinbyeo). The study reported that post-application of JA after NaCl treatment (i) reduced uptake of Na^+^ (ii) slightly increased uptake of K^+^ and (iii) reduced salt inhibition of dry mass production. Leaf water potential, leaf photosynthetic rate, and maximum quantum yield of photosystem II (PSII) also remarkably recovered when JA was applied 24 h after the salt stress [101]. These studies indicated that accumulation of jasmonates could improve salt tolerance in rice.

However, recent evidence suggested that suppression of jasmonates signaling is important for the enhancement of salt tolerance in rice. Transgenic rice plants overexpressing the repressor gene *OsJAZ9* accumulated a higher level of proline and grew better than the wild type plants (*Oryza sativa* subsp. *japonica* cv Zhonghua 11) under high salinity conditions [10]. Further studies suggested that *OsJAZ9* confers salt tolerance possibly by regulating the Na^+^/K^+^ ratio. OsJAZ9 (together with NINJA) interacted with OsbHLH062 and inhibited OsbHLH062-mediated transcription activation, which regulates the expression of key genes involved in ions homeostasis, such as *OsSKC1*, *OsHAK21*, and *OsHAK27* [102]. A low Na^+^/K^+^ ratio is correlated with salt tolerance in rice [103,104]. The Na^+^/K^+^ ratio in the *OsJAZ9*-overexpression plants was lower than that in wild type and *OsJAZ9*-suppression plants under salt stress conditions [102]. In addition, the ability to maintain meristem activities is important for the plant to continue to grow in high salt conditions. OsJAZ9 and OsJAZ11 were reported to form a ternary complex with the RSS3 (RICE SALT SENSITIVE3) and OsbHLH094 and suppress OsbHLH094-mediated transcription activation, specifically in the root tip, to maintain the meristem activities and root elongation in salinity conditions. Loss of function of RSS3 failed to interact with OsbHLH094 and the expression of a significant portion of JA-responsive genes was upregulated in *rss3*, resulted in a defect in root development under salt stress conditions [105]. Other pieces of evidence come from the finding that the transgenic plants overexpressing the *CYP94C2b* gene (Os12g0150200), which is involved in the deactivation of JA-Ile, were less sensitive to salt stress than the wild type (*Oryza sativa* subsp. *japonica* cv Nipponbare) [106,107]. Overexpression of *CYP94C2b* resulted in lower sensitivity to JA and a delay in salinity-induced leaf senescence [106]. Notably, comparative evaluation of nine salt-tolerant rice varieties and transgenic rice lines carrying constitutively expressed genes/QTLs that are potentially involved in salt tolerance revealed that the levels of salt tolerance in the *CYP94C2b*–overexpressing plants were comparable with those in Nona Bokra and Pokkali, the two most salt-tolerant varieties [107]. Besides *CYP94C2b*, other members of the *CYP94* gene family are under investigation [108]. Salt stress induces expression of several genes, such as *CYP94B5, CYP94D7*, and *CYP94D9*. In addition, CYP94B5 has been shown to oxidize JA-Ile into 12OH-JA-Ile in vitro [108]. However, the contribution of *CYP94B5* in salt response requires further investigation. These results suggested that the deactivation of JA-Ile is one of the key determinants for viability under salinity conditions. Studies on the JA biosynthesis rice mutants (*cpm2* and *hebiba*) and the corresponding wild type (*Oryza sativa* subsp. *japonica* cv Nihonmasari), further support this argument. These mutants exhibited less sensitivity to salt stress, accumulated smaller amounts of Na^+^ ions in their leaves, and showed better scavenging of reactive oxygen species (ROS) under salt stress [109]. All these results are in line with the conclusion that jasmonates have a negative role in salt tolerance in rice.

### 4.2. Roles of Jasmonates in Drought Stress

Drought is another constraint inhibiting rice production. Similar to salt response, the precise role of jasmonates signaling in drought-tolerant rice remains controversial. Some studies have reported that induced JA signaling enhances rice tolerance to drought. Seo et al. (2011) [110] identified a rice basic helix-loop-helix (bHLH) domain gene, *OsbHLH148*, conferring drought tolerance as a component of the jasmonate signaling module in rice. OsbHLH148 physically interacts with the OsJAZs protein, such as OsJAZ12 (originally referred to as OsJAZ1), as demonstrated in a yeast-two-hybrid and pull-down assay. Furthermore, OsJAZ12 also interacted with the OsCOI1a protein in the presence of coronatine and was degraded by a SCF^OsCOI1^ complex-mediated 26S proteasome. Based on these findings, a signaling module was proposed that in normal conditions, OsJAZ proteins inhibit the transcription of OsbHLH148-mediated, stress-responsive genes (such as *OsDREB1s*). Drought stress induces the accumulation of jasmonates promoting degradation of OsJAZ proteins and allows induction of OsbHLH148-mediated responsive genes leading to drought tolerance [110]. One of the JA/salt-responsive genes, *JIOsPR10*, was found to increase both salt and drought tolerance when overexpressed in rice [78]. However, it is unclear whether *JIOsPR10* is an *OsbHLH148*-mediated responsive gene. In agreement with these reports, the study of Fu et al. (2017) [111] supported the role of OsJAZ proteins as the negative regulator in JA signaling involved in drought resistance. As demonstrated for the case of *OsJAZ1*, rice plants overexpressing *OsJAZ1* (in the genetic background of *Oryza sativa* subsp. *japonica* cv Zhonghua 11) showed increased susceptibility to drought at both seedling and reproductive stages, with reduced relative leaf water and enhanced the degree of leaf rolling. Interestingly, induction of *OsbHLH148* and *OsbHLH148*-downstream genes were abolished in *OsJAZ1_OE* plants [111]. Although OsJAZ1 could not interact directly with OsbHLH148 [110], it was found that JAZ proteins act with other JAZs by forming homo/hetero-dimers through their conserved ZIM domain [94], suggesting that transcriptional activation activities of OsbHLH148 could be inhibited by dimerization between OsJAZ1 and OsJAZ12. Another possibility is that redundancy of OsJAZ1 inhibited jasmonates signaling, hence preventing other OsJAZ proteins from degradation. Indeed, *OsJAZ1_OE* plants were hyposensitive to MeJA. Consistent with these results, *osjaz1* mutant showed hypersensitivity to MeJA and increased tolerant to drought [111]. Collectively, these results suggested a positive role of jasmonates signaling in rice drought resistance. 

On the contrary, a recent study using the JA biosynthesis mutant, *cpm2,* showed that the mutant displayed a higher tolerance to drought stress [112]. Under severe drought conditions, *cpm2* mutant showed better physiological responses, such as higher stomatal conductance and increased water use efficiency as compared to the wild type. Notably, roots of *cpm2* were better developed compared to the wild type under both control and drought stress conditions. Analysis of root proteome suggested increased energy metabolism (i.e., increased mobilization of resources) and reactive oxygen species scavenging in *cpm2* under drought. Additionally, various proteins involved in secondary metabolism, cell growth and cell wall synthesis were also more abundant in *cpm2* roots [112]. Thus, these results suggested that jasmonates might have a negative role in drought response in rice.

### 4.3. Roles of Jasmonates in Cold and Freezing Tolerance

Rice and many other agriculturally important crops are chilling sensitive and unable to survive freezing temperatures. Recent studies in Arabidopsis have revealed the roles for JAs as positive regulators of cold and freezing tolerance [113]. Firstly, exposure to cold rapidly elevates endogenous jasmonates levels by inducing JA biosynthesis genes such as *LOX1*, *AOS1*, *AOC1*, and *JAR1*. Expectedly, treatment with exogenous JA enhanced freezing tolerance in Arabidopsis. Furthermore, Arabidopsis mutants deficient in JA biosynthesis or signaling (i.e., *lox2*, *aos, jar1*, and *coi1*) displayed increased sensitivity to freezing stress relative to wild type plants [113]. It was found that jasmonates regulate cold tolerance through the *INDUCER OF CBF EXPRESSION 1 (ICE1). ICE1* is a positive regulator of cold tolerance by activating *C-REPEAT BINDING FACTOR*s (*CBF*s), which encode *AP2/ERF* family TFs. CBFs, by binding to the C-repeat /dehydration-responsive element, activate *COLD REGULATED* genes with roles in stress protection [114]. The repressor JAZ protein was found to participate in this regulation as a repressor of *ICE*-mediated activation of cold-responsive genes in normal growth conditions to prevent non-specific activation of cold stress responses. Under cold stress, increased jasmonates levels trigger COI1-mediated degradation of JAZs, and this releases ICEs from repression allowing the activation of downstream genes [113]. Cold stress also induces the production of JA biosynthesis in rice [10], suggesting that JA is also involved in cold tolerance in this crop. Two genes *OsICE1* and *OsICE2* were identified as functional orthologues of *ICE1* in Arabidopsis. Overexpression of *OsICEs* conferred enhanced cold tolerance in transgenic Arabidopsis [115]. So far, which OsJAZ protein it is that interacts with OsICE1 and OsICE2 is not yet identified. A recent study exploited the natural variation in the rice collection to identify the *HAN1* gene that confers chill tolerance in rice and allows the adaptation to a temperate climate [116]. *HAN1* encodes an oxidase that catalyzes the conversion of biologically active jasmonoyl-L-isoleucine (JA-Ile) to the inactive form 12-hydroxy-JA-Ile (12OH-JA-Ile) and fine-tunes the JA-mediated chill response. The temperate *japonica* rice cultivar “02428” has a lower expression of *HAN1*, which causes it to maintain higher JA-Ile levels and higher gene expressions in the *CBF/DREB1*-dependent pathway, thus conferring greater chill tolerance compared with the *indica* rice cultivar Teqing [116]. Together, these results suggested the positive role of JA in tolerance to freezing stress in rice.

### 4.4. Roles of Jasmonates in Nutrient Deficiency

Beside important roles in drought, salt, and cold stress, in recent years, many reports have announced the involvement of jasmonates in response to nutrients starvation or modulated nutrient status in unfavorable conditions. Kobayashi et al. [117] reported a rapid induction of genes involved in JA biosynthesis and signaling in the very early stages of iron deficiency in rice roots (*Oryza sativa* subsp. *japonica* cv Tsukinohikari). Accordingly, the endogenous level of JA and JA-Ile were increased rapidly in rice roots growth in Fe-inefficient conditions [117]. A comparison with the JA-deficient *cpm2* mutant revealed that, in the wild type plant, jasmonates repress the expression of many iron deficiency-inducible genes involved in iron uptake and translocation under iron sufficiency, but this repression is partly canceled under an early stage of iron deficiency [117], although the molecular basis of jasmonates signaling and the role of the OsJAZ proteins in iron deficiency response are elusive. However, based on these findings, a putative signaling module responding to JAZ proteins and the activator transcription factors likely exists, in which OsJAZ proteins act as a repressor of the activator transcription factor-mediated regulation of iron-deficiency-responsive genes. In fact, three bHLH transcription factors, *OsIRO2, OsIRO3,* and *OsbHLH133* have been identified to be involved in the Fe deficiency response in rice [118]. Whereas, many *OsJAZ* genes have also been found induced in rice roots under macronutrients (N, P, K) and micronutrients (such as Cu, Fe, …)-deficient conditions [119]. It would be interesting to find out whether or not these basic helix-loop-helix transcription factors could interact with the OsJAZs protein.

Another study implied the roles of jasmonate signaling in nitrogen uptake and allocation in rice (*Oryza sativa* subsp. *japonica* cv Shishoubaimao) [120]. Exogenous applications of MeJA to rice seedlings led to significantly reduced N uptake in roots and reduced translocation of recently-absorbed labeled ^15^N from roots to leaves, likely occurring as a result of down-regulation of the glutamine synthetase cytosolic isozyme 1-2 and ferredoxin-nitrite reductase. Shoot MeJA treatment resulted in the remobilization of endogenous unlabeled ^14^N from leaves to roots, and root MeJA treatment also increased ^14^N accumulation in roots but did not affect ^14^N accumulation in leaves of rice. Additionally, proteomic and gene expression analysis showed that JA-mediated plastid disassembly and dehydrogenases *GDH2* up-regulation contribute to N release in leaves to support the production of defensive proteins/compounds under an N-limited condition. Collectively, these results indicate that JA signaling mediates large-scale systemic changes in N uptake and allocation in rice plant [120].

## 5. Conclusion and Future Perspectives

In the past decades, significant progress has been achieved in our understanding of jasmonate signaling and its actions in rice. As expected, the functions of JA in rice are versatile. It is involved in every aspect of rice plant development from seed-to-seed, as well as in mediating responses to abiotic and biotic stresses. 

Despite these recent breakthroughs, our knowledge on JA signaling and its functions in rice are still modest compared to the achievements in Arabidopsis. So far, reverse genetic approaches aimed at testing the roles of rice homologs of Arabidopsis genes have played a prominent role in deciphering the JA signaling in rice plants. However, future research in this crop could also bring new insights specific for the monocotyledonous group. For instance, the existence of three receptor proteins suggesting an increasing number of possible COI-JAZ interactions may be a strategy to help fine-tune downstream JA signaling in response to specific stressors in rice. Furthermore, differences in morphology and pathogen-host specificities between the two plant systems suggest that JA biosynthetic or signaling components particular to rice and other monocot plants may exist. 

There are several questions that remain challenges in the jasmonates field: (1) how can plants discriminate the type of external stress signals or developmental stimuli specifically, and lead to the activation of JA biosynthesis and signaling? (2) By which mechanism does a single bioactive JA hormone regulate a myriad of physiological processes and provide specificity in the response? The answer for the first question might be sought in the class of pattern recognition receptors (PRRs) which directly perceive extracellular signals. For example, one member of the leucine-rich repeat receptor-like kinases (OsLRR-RLK1) has been shown to initiate defense response and positively regulate the level of herbivore-induced JA [66]. How the plant perceives abiotic signals is largely unknown. Therefore, the window for studying the mechanism of the plant in perceiving stress signals is largely open. Regarding the second question, the hypothesis is based on the tight regulation by homeostasis of regulatory modules containing repressors, activators, the ligand JA-Ile, and the SCFCOI1-JAZ-coreceptor complex, as well as the spatio-temporal distribution of components involved [5,96,121,122,123]. In rice, this homeostasis status is even more challenging to dissect, due to both the higher number of components involved and the fact that most of them are not yet characterized. However, some pieces of the picture have begun to appear, as shown in Figure 1. The work of Kang et al. (2005) [101] is a good example demonstrating the importance of right timing regulation, with the finding that only rice plant pre-treatment with JA, but not during or after, exposure to salt stress gave benefit to the plant, even though the actual role of JA in mediating salt and drought response is still unclear [112]. Thus, in the near future, more endeavors are still needed to identify the new regulators with a special focus on the homeostasis and spatial-temporal signature of JA signaling in rice plants.

## Figures and Tables

**Table 1:** Rice pathogens and pests whose parasitism is reduced by Jasmonates-triggered immunity.

Organism	Common name	Lifestyle	Reference
**Pathogenic bacteria** *Xanthomonas oryzae*	Bacterial blight	Obligate biotroph	[47,48,57,58,59]
**Nematodes** *Meloidogyne graminicola*	Root-knot nematode	Obligate biotroph	[60]
**Leaf/root chewing insects** *Chilo suppressalis* *Cnaphalocrocis medinalis* *Spodoptera mauritia* *Spodoptera frugiperda* *Diabrotica balteat* *Lissorhoptrus oryzophilus*	Rice striped stem borer (SSB) Rice leaffolder (LF) Lawn armyworms Fall armyworm Cucumber beetle Rice water weevil	Obligate biotroph Obligate biotroph Obligate biotroph Obligate biotroph Obligate biotroph Obligate biotroph	[61,62,63,64,65,66,67] [63,64,65] [68] [69] [70] [70]
**Piercing-sucking insects** *Nilaparvata lugens* *Sogatella furcifera*	Rice brown planthopper (BPH) White-backed planthopper	Obligate biotroph Obligate biotroph	[61,62,63,65,71,72] [73]
**Virus** *Rice ragged stunt virus* *Rice black streaked dwarf virus*	Rice ragged stunt virus Rice black streaked dwarf virus	Obligate biotroph Obligate biotroph	[74] [75]
**Parasitic plant** *Striga hermonthica*	Root hemiparasite witchweed	Hemiparasite	[76]
**Fungi** *Magnaporthe oryzae*	Rice blast fungus	Hemibiotroph	[30,77,78,79,80,81]
**Crustaceans** *Armadillidium vulgare*	Pill-bug	Detritivore	[82]
**Detritivores insect** *Bradysia spp. (Diptera: Sciaridae)*	Fungus gnats	Detritivore	This study

## Supplementary Materials

The following are available online at https://www.mdpi.com/2223-7747/8/9/339/s1, Table S1: Mutant and transgenic plants in jasmonates biosynthesis and signaling in rice.

## References

[1] Campos M.L., Kang J.H., Howe G.A. (2014). Jasmonate-triggered plant immunity. J. Chem. Ecol..

[2] Hou X., Ding L., Yu H. (2013). Crosstalk between GA and JA signaling mediates plant growth and defense. Plant Cell Rep..

[3] Kazan K. (2015). Diverse roles of jasmonates and ethylene in abiotic stress tolerance. Trends Plant Sci..

[4] Okada K., Abe H., Arimura G. (2015). Jasmonates induce both defense responses and communication in monocotyledonous and dicotyledonous plants. Plant Cell Physiol..

[5] Riemann M., Dhakarey R., Hazman M., Miro B., Kohli A., Nick P. (2015). Exploring jasmonates in the hormonal network of drought and salinity responses. Front. Plant Sci..

[6] Zhang L., Zhang F., Melotto M., Yao J., He S.Y. (2017). Jasmonate signaling and manipulation by pathogens and insects. J. Exp. Bot..

[7] Huang H., Liu B., Liu L., Song S. (2017). Jasmonate action in plant growth and development. J. Exp. Bot..

[8] Qi J., ul Malook S., Shen G., Gao L., Zhang C., Li J., Zhang J., Wang L., Wu J. (2018). Current understanding of maize and rice defense against insect herbivores. Plant Divers..

[9] Lee H.Y., Seo J.S., Cho J.H., Jung H., Kim J.K., Lee J.S., Rhee S., Do Choi Y. (2013). Oryza sativa COI homologues restore jasmonate signal transduction in Arabidopsis coi1-1 mutants. PLoS ONE.

[10] Ye H., Du H., Tang N., Li X., Xiong L. (2009). Identification and expression profiling analysis of TIFY family genes involved in stress and phytohormone responses in rice. Plant Mol. Biol..

[11] Dhakarey R., Kodackattumannil Peethambaran P., Riemann M. (2016). Functional analysis of jasmonates in rice through mutant approaches. Plants.

[12] Poudel A.N., Holtsclaw R.E., Kimberlin A., Sen S., Zeng S., Joshi T., Lei Z., Sumner L.W., Singh K., Matsuura H. (2019). 12-Hydroxy-jasmonoyl-L-isoleucine is an active jasmonate that signals through CORONATINE INSENSITIVE 1 and contributes to the wound response in Arabidopsis. Plant Cell Physiol..

[13] Monte I., Ishida S., Zamarreño A.M., Hamberg M., Franco-Zorrilla J.M., García-Casado G., Gouhier-Darimont C., Reymond P., Takahashi K., García-Mina J.M. (2018). Solano, R. Ligand-receptor co-evolution shaped the jasmonate pathway in land plants. Nat. Chem. Biol..

[14] Svyatyna K., Riemann M. (2012). Light-dependent regulation of the jasmonate pathway. Protoplasma.

[15] Lyons R., Manners J.M., Kazan K. (2013). Jasmonate biosynthesis and signaling in monocots: A comparative overview. Plant Cell Rep..

[16] Liu Z., Zhang S., Sun N., Liu H., Zhao Y., Liang Y., Zhang L., Han Y. (2015). Functional diversity of jasmonates in rice. Rice.

[17] Park J.H., Halitschke R., Kim H.B., Baldwin I.T., Feldmann K.A., Feyereisen R. (2002). A knock-out mutation in *ALLENE OXIDE SYNTHASE* results in male sterility and defective wound signal transduction in Arabidopsis due to a block in jasmonic acid biosynthesis. Plant J..

[18] Caldelari D., Wang G., Farmer E.E., Dong X. (2011). Arabidopsis *lox3 lox4* double mutants are male sterile and defective in global proliferative arrest. Plant Mol. Biol..

[19] Thines B., Mandaokar A., Browse J. (2013). Characterizing jasmonate regulation of male fertility in Arabidopsis. Methods Mol. Biol..

[20] Nakata M., Ohme-Takagi M. (2013). Two bHLH-type transcription factors, JA-associated MYC2-like 2 and JAM3, are transcriptional repressors and affect male fertility. Plant Signal. Behav..

[21] Shih C.F., Hsu W.H., Peng Y.J., Yang C.H. (2014). The NAC-like gene *ANTHER INDEHISCENCE FACTOR* acts as a repressor that controls anther dehiscence by regulating genes in the jasmonate biosynthesis pathway in Arabidopsis. J. Exp. Bot..

[22] Li L., Zhao Y., McCaig B.C., Wingerd B.A., Wang J., Whalon M.E., Pichersky E., Howe G.A. (2004). The tomato homolog of *CORONATINE-INSENSITIVE1* is required for the maternal control of seed maturation, jasmonate-signaled defense responses, and glandular trichome development. Plant Cell.

[23] Dobritzsch S., Weyhe M., Schubert R., Dindas J., Hause G., Kopka J., Hause B. (2015). Dissection of jasmonate functions in tomato stamen development by transcriptome and metabolome analyses. BMC Biol..

[24] Schubert R., Dobritzsch S., Gruber C., Hause G., Athmer B., Schreiber T., Marillonnet S., Okabe Y., Ezura H., Acosta I.F. (2019). Tomato *MYB21* acts in ovules to mediate jasmonate-eegulated fertility. Plant Cell.

[25] Li Y., Jiang J., Du M.L., Li L., Wang X.L., Li X.B. (2013). A cotton gene encoding *MYB*-like transcription factor is specifically expressed in pollen and is involved in regulation of late anther/pollen development. Plant Cell Physiol..

[26] Stumpe M., Göbel C., Faltin B., Beike A.K., Hause B., Himmelsbach K., Bode J., Kramell R., Wasternack C., Frank W. (2010). The moss *Physcomitrella patens* contains cyclopentenones but no jasmonates: Mutations in allene oxide cyclase lead to reduced fertility and altered sporophyte morphology. New Phytol..

[27] Acosta I.F., Laparra H., Romero S.P., Schmelz E., Hamberg M., Mottinger J.P., Moreno M.A., Dellaporta S.L. (2009). *tasselseed1* is a lipoxygenase affecting jasmonic acid signaling in sex determination of maize. Science.

[28] Yan Y., Christensen S., Isakeit T., Engelberth J., Meeley R., Hayward A., Emery R.J.N., Kolomiets M.V. (2012). Disruption of *OPR7* and *OPR8* reveals the versatile functions of jasmonic acid in maize development and defense. Plant Cell.

[29] Riemann M., Müller A., Korte A., Furuya M., Weiler E.W., Nick P. (2003). Impaired induction of the jasmonate pathway in the rice mutant hebiba. Plant Physiol..

[30] Riemann M., Haga K., Shimizu T., Okada K., Ando S., Mochizuki S., Nishizawa Y., Yamanouchi U., Nick P., Yano M. (2013). Identification of rice *ALLENE OXIDE CYCLASE* mutants and the function of jasmonate for defence against Magnaporthe oryzae. Plant J..

[31] Cai Q., Yuan Z., Chen M., Yin C., Luo Z., Zhao X., Liang W., Hu J., Zhang D. (2014). Jasmonic acid regulates spikelet development in rice. Nat. Commun..

[32] Biswas K.K., Neumann R., Haga K., Yatoh O., Iino M. (2003). Photomorphogenesis of rice seedlings: A mutant impaired in phytochrome-mediated inhibition of coleoptile growth. Plant Cell Physiol..

[33] Hibara K.I., Isono M., Mimura M., Sentoku N., Kojima M., Sakakibara H., Kitomi Y., Yoshikawa T., Itoh J.I., Nagato Y. (2016). Jasmonate regulates juvenile-to-adult phase transition in rice. Development.

[34] Liao L., Shi C.H., Zeng D.D., Jin X.L., Wu J.G. (2015). Morphogenesis and molecular basis on the *UNCLOSED GLUMES*, a novel mutation related to the floral organ of rice. Plant Mol. Biol. Rep..

[35] Li X., Wang Y., Duan E., Qi Q., Zhou K., Lin Q., Wang D., Wang Y., Long W., Zhao Z. (2018). *OPEN GLUME1*: A key enzyme reducing the precursor of JA, participates in carbohydrate transport of lodicules during anthesis in rice. Plant Cell Rep..

[36] Xiao Y., Chen Y., Charnikhova T., Mulder P.P.J., Heijmans J., Hoogenboom A., Agalou A., Michel C., Morel J.B., Dreni L. (2014). *OsJAR1* is required for JA-regulated floret opening and anther dehiscence in rice. Plant Mol. Biol..

[37] You X., Zhu S., Zhang W., Zhang J., Wang C., Jing R., Chen W., Wu H., Cai Y., Feng Z. (2019). *OsPEX5* regulates rice spikelet development through modulating jasmonic acid biosynthesis. New Phytol..

[38] Kim E.H., Kim Y.S., Park S.H., Koo Y.J., Choi Y.D., Chung Y.Y., Lee I.J., Kim J.K. (2009). Methyl jasmonate reduces grain yield by mediating stress signals to alter spikelet development in rice. Plant Physiol..

[39] Lee S.H., Sakuraba Y., Lee T., Kim K.W., An G., Lee H.Y., Paek N.C. (2015). Mutation of *Oryza sativa CORONATINE INSENSITIVE 1b (OsCOI1b*) delays leaf senescence. J. Integr. Plant Biol..

[40] Yang D.L., Yao J., Mei C.S., Tong X.H., Zeng L.J., Li Q., Xiao L.T., Sun T.P., Li J., Deng X.W. (2012). Plant hormone jasmonate prioritizes defense over growth by interfering with gibberellin signaling cascade. Proc. Natl. Acad. Sci. USA.

[41] Chini A., Gimenez-Ibanez S., Goossens A., Solano R. (2016). Redundancy and specificity in jasmonate signalling. Curr. Opin. Plant Biol..

[42] Cheng H., Song S., Xiao L., Soo H.M., Cheng Z., Xie D., Peng J. (2009). Gibberellin acts through jasmonate to control the expression of *MYB21, MYB24,* and *MYB57* to promote stamen filament growth in Arabidopsis. PLoS Gene..

[43] Qi T., Huang H., Song S., Xie D. (2015). Regulation of jasmonate-mediated stamen development and seed production by a bHLH-MYB Complex in Arabidopsis. Plant Cell.

[44] Hori Y., Kurotani K., Toda Y., Hattori T., Takeda S. (2014). Overexpression of the JAZ factors with mutated jas domains causes pleiotropic defects in rice spikelet development. Plant Signal. Behav..

[45] Hakata M., Muramatsu M., Nakamura H., Hara N., Kishimoto M., Iida-Okada K., Kajikawa M., Imai-Toki N., Toki S., Nagamura Y. (2017). Overexpression of *TIFY* genes promotes plant growth in rice through jasmonate signaling. Biosci. Biotechnol. Biochem..

[46] Li X., Guo Z., Lv Y., Cen X., Ding X., Wu H., Li X., Huang J., Xiong L. (2017). Genetic control of the root system in rice under normal and drought stress conditions by genome-wide association study. PLoS Genet..

[47] Kashihara K., Onohata T., Okamoto Y., Uji Y., Mochizuki S., Akimitsu K., Gomi K. (2019). Overexpression of *OsNINJA1* negatively affects a part of *OsMYC2*-mediated abiotic and biotic responses in rice. J. Plant Physiol..

[48] Uji Y., Taniguchi S., Tamaoki D., Shishido H., Akimitsu K., Gomi K. (2016). Overexpression of *OsMYC2* Results in the up-regulation of early JA-responsive genes and bacterial blight resistance in rice. Plant Cell Physiol..

[49] To T.M.H., Nguyen T.H., Dang T.M.N., Nguyen H.N., Bui X.T., Lavarenne J., Phung T.P.N., Gantet P., Lebrun M., Bellafiore S. Unraveling the genetic elements involved in shoot and root growth regulation by jasmonate in rice using a genome-wide association study. Rice.

[50] Dombrecht B., Xue G.P., Sprague S.J., Kirkegaard J.A., Ross J.J., Reid J.B., Fitt G.P., Sewelam N., Schenk P.M., Manners J.M. (2007). *MYC2* differentially modulates diverse jasmonate-dependent functions in Arabidopsis. Plant Cell.

[51] Besseau S., Hoffmann L., Geoffroy P., Lapierre C., Pollet B., Legrand M. (2007). Flavonoid accumulation in Arabidopsis repressed in lignin synthesis affects auxin transport and plant growth. Plant Cell.

[52] Brown D.E., Rashotte A.M., Murphy A.S., Normanly J., Tague B.W., Peer W.A., Taiz L., Muday G.K. (2001). Flavonoids act as negative regulators of auxin transport in vivo in Arabidopsis. Plant Physiol..

[53] Ogawa S., Kawahara-Miki R., Miyamoto K., Yamane H., Nojiri H., Tsujii Y., Okada K. (2017). *OsMYC2* mediates numerous defence-related transcriptional changes via jasmonic acid signalling in rice. Biochem. Biophys. Res. Commun..

[54] Yamane H. (2013). Biosynthesis of phytoalexins and regulatory mechanisms of it in Rice. Biosci. Biotechnol. Biochem..

[55] Miyamoto K., Enda I., Okada T., Sato Y., Watanabe K., Sakazawa T., Yumoto E., Shibata K., Asahina M., Iino M. (2016). Jasmonoyl-l-isoleucine is required for the production of a flavonoid phytoalexin but not diterpenoid phytoalexins in ultraviolet-irradiated rice leaves. Biosci. Biotechnol. Biochem..

[56] Farmer E.E., Ryan C.A. (1990). Interplant communication: Airborne methyl jasmonate induces synthesis of proteinase inhibitors in plant leaves. Proc. Natl. Acad. Sci. USA.

[57] Yamada S., Kano A., Tamaoki D., Miyamoto A., Shishido H., Miyoshi S., Taniguchi S., Akimitsu K., Gomi K. (2012). Involvement of *OsJAZ8* in jasmonate-induced resistance to bacterial blight in rice. Plant Cell Physiol..

[58] Yoo Y., Park J.C., Cho M.H., Yang J., Kim C.Y., Jung K.H., Jeon J.S., An G., Lee S.W. (2018). Lack of a cytoplasmic RLK, required for ROS homeostasis, induces strong resistance to bacterial leaf blight in rice. Front. Plant Sci..

[59] Hui S., Hao M., Liu H., Xiao J., Li X., Yuan M., Wang S. (2019). The group I *GH3* family genes encoding *JA-ILE SYNTHETASE* act as positive regulator in the resistance of rice to *Xanthomonas oryzae pv. oryzae*. Biochem. Biophys. Res. Commun..

[60] Nahar K., Kyndt T., De Vleesschauwer D., Höfte M., Gheysen G. (2011). The jasmonate pathway is a key player in systemically induced defense against root knot nematodes in rice. Plant Physiol..

[61] Qi J., Zhou G., Yang L., Erb M., Lu Y., Sun X., Cheng J., Lou Y. (2011). The Chloroplast-localized Phospholipases D 4 and 5 regulate herbivore-induced direct and indirect defenses in rice. Plant Physiol..

[62] Zhou G., Qi J., Ren N., Cheng J., Erb M., Mao B., Lou Y. (2009). Silencing *OsHI-LOX* makes rice more susceptible to chewing herbivores, but enhances resistance to a phloem feeder. Plant J..

[63] Ye M., Luo S.M., Xie J.F., Li Y.F., Xu T., Liu Y., Song Y.Y., Zhu-Salzman K., Zeng R.S. (2012). Silencing *COI1* in rice increases susceptibility to chewing insects and impairs inducible defense. PLoS ONE.

[64] Li R., Afsheen S., Xin Z., Han X., Lou Y. (2013). *OsNPR1* negatively regulates herbivore-induced JA and ethylene signaling and plant resistance to a chewing herbivore in rice. Physiol. Plant.

[65] Wang Q., Li J., Hu L., Zhang T., Zhang G., Lou Y. (2013). *OsMPK3* positively regulates the JA signaling pathway and plant resistance to a chewing herbivore in rice. Plant Cell Rep..

[66] Hu L., Ye M., Kuai P., Ye M., Erb M., Lou Y. (2018). OsLRR-RLK1, an early responsive leucine-rich repeat receptor-like kinase, initiates rice defense responses against a chewing herbivore. New Phytol..

[67] Liu X., Li J., Xu L., Wang Q., Lou Y. (2018). Expressing *OsMPK4* impairs plant growth but enhances the resistance of rice to the striped stem borer *Chilo suppressalis*. Int. J. Mol. Sci..

[68] Fukumoto K., Alamgir K., Yamashita Y., Mori I.C., Matsuura H., Galis I. (2013). Response of rice to insect elicitors and the role of *OsJAR1* in wound and herbivory-induced JA-Ile accumulation. J. Integr. Plant Biol..

[69] Ye M., Glauser G., Lou Y., Erb M., Hu L. (2019). Molecular dissection of early defense signaling underlying volatile-mediated defense regulation and herbivore resistance in rice. Plant Cell.

[70] Lu J., Robert C.A.M., Riemann M., Cosme M., Mène-Saffrané L., Massana J., Stout M.J., Lou Y., Gershenzon J., Erb M. (2015). Induced jasmonate signaling leads to contrasting effects on root damage and herbivore performance. Plant Physiol..

[71] Guo H.M., Li H.C., Zhou S.R., Xue H.W., Miao X.X. (2014). Cis-12-oxo-phytodienoic acid stimulates rice defense response to a piercing-sucking insect. Mol. Plant.

[72] Wang M., Yang D., Ma F., Zhu M., Shi Z., Miao X. (2019). *OsHLH61-OsbHLH96* influences rice defense to brown planthopper through regulating the pathogen-related genes. Rice.

[73] Li P., Liu H., Li F., Liao X., Ali S., Hou M. (2018). A virus plays a role in partially suppressing plant defenses induced by the viruliferous vectors. Sci. Rep..

[74] Zhang C., Ding Z., Wu K., Yang L., Li Y., Yang Z., Shi S., Liu X., Zhao S., Yang Z. (2016). Suppression of jasmonic acid-mediated defense by viral-inducible *MicroRNA319* facilitates virus infection in rice. Mol. Plant.

[75] He Y., Zhang H., Sun Z., Li J., Hong G., Zhu Q., Zhou X., MacFarlane S., Yan F., Chen J. (2017). Jasmonic acid-mediated defense suppresses brassinosteroid-mediated susceptibility to *Rice black streaked dwarf virus* infection in rice. New Phytol..

[76] Mutuku J.M., Yoshida S., Shimizu T., Ichihashi Y., Wakatake T., Takahashi A., Seo M., Shirasu K. (2015). The WRKY45-dependent signaling pathway is required for resistance against *Striga hermonthica* parasitism. Plant Physiol..

[77] Patkar R.N., Benke P.I., Qu Z., Chen Y.Y.C., Yang F., Swarup S., Naqvi N.I. (2015). A fungal monooxygenase-derived jasmonate attenuates host innate immunity. Nat. Chem. Biol..

[78] Wu J., Kim S.G., Kang K.Y., Kim J.G., Park S.R., Gupta R., Kim Y.H., Wang Y., Kim S.T. (2016). Overexpression of a Pathogenesis-Related protein 10 enhances biotic and abiotic stress tolerance in rice. Plant Pathol. J..

[79] Jiang C.J., Liu X.L., Liu X.Q., Zhang H., Yu Y.J., Liang Z.W. (2017). Stunted growth caused by blast disease in rice seedlings is associated with changes in phytohormone signaling pathways. Front. Plant Sci..

[80] Zhang X., Bao Y., Shan D., Wang Z., Song X., Wang Z., Wang J., He L., Wu L., Zhang Z. (2018). *Magnaporthe oryzae* induces the expression of a MicroRNA to suppress the immune response in rice. Plant Physiol..

[81] Tezuka D., Kawamata A., Kato H., Saburi W., Mori H., Imai R. (2019). The rice ethylene response factor *OsERF83* positively regulates disease resistance to *Magnaporthe oryzae*. Plant Physiol. Biochem..

[82] Farmer E.E., Dubugnon L. (2009). Detritivorous crustaceans become herbivores on jasmonate-deficient plants. Proc. Natl. Acad. Sci. USA.

[83] Yan Y., Huang P.C., Borrego E., Kolomiets M. (2014). New perspectives into jasmonate roles in maize. Plant Signal Behav..

[84] Staswick P.E., Yuen G.Y., Lehman C.C. (1998). Jasmonate signaling mutants of Arabidopsis are susceptible to the soil fungus *Pythium irregulare*. Plant J..

[85] Cloyd R.A. (2015). Ecology of fungus gnats (*Bradysia* spp.) in greenhouse production systems associated with disease-interactions and alternative management strategies. Insects.

[86] Zhang H., He Y., Tan X., Xie K., Li L., Hong G., Li J., Cheng Y., Yan F., Chen J. (2019). The dual effect of the brassinosteroid pathway on *Rice Black Streaked Dwarf Virus* infection by modulating the peroxidase-mediated oxidative burst and plant defense. Mol. Plant Microbe Interact..

[87] Senthil-Nathan S. (2019). Effect of methyl jasmonate (MeJA)-induced defenses in rice against the rice leaffolder *Cnaphalocrocis medinalis* (Guenèe) (*Lepidoptera: Pyralidae*). Pest Manag. Sci..

[88] Jisha V., Dampanaboina L., Vadassery J., Mithöfer A., Kappara S., Ramanan R. (2015). Overexpression of an AP2/ERF type transcription factor *OsEREBP1* confers biotic and abiotic stress tolerance in rice. PLoS ONE.

[89] Giri M.K., Gautam J.K., Rajendra Prasad V.B., Chattopadhyay S., Nandi A.K. (2017). Rice *MYC2* (*OsMYC2*) modulates light-dependent seedling phenotype, disease defence but not ABA signalling. J. Biosci..

[90] Chini A., Fonseca S., Fernández G., Adie B., Chico J.M., Lorenzo O., García-Casado G., López-Vidriero I., Lozano F.M., Ponce M.R. (2007). The JAZ family of repressors is the missing link in jasmonate signalling. Nature.

[91] Thines B., Katsir L., Melotto M., Niu Y., Mandaokar A., Liu G., Nomura K., He S.Y., Howe G.A., Browse J. (2007). JAZ repressor proteins are targets of the SCF^(COI1)^ complex during jasmonate signalling. Nature.

[92] Torres-Vera R., Garcıa J.M., Pozo M.J., Lopez-Raez J.A. (2014). Do strigolactones contribute to plant defence?. Mol. Plant Pathol..

[93] Lahari Z., Ullah C., Kyndt T., Gershenzon J., Gheysen G. (2019). Strigolactones enhance root-knot nematode (*Meloidogyne graminicola*) infection in rice by antagonizing the jasmonate pathway. New Phytol..

[94] Pauwels L., Goossens A. (2011). The JAZ Proteins: A crucial interface in the jasmonate signaling cascade. Plant Cell.

[95] Huot B., Yao J., Montgomery B.L., He S.Y. (2014). Growth-defense tradeoffs in plants: A balancing act to optimize fitness. Mol. Plant.

[96] Campos M.L., Yoshida Y., Major I.T., de Oliveira Ferreira D., Weraduwage S.M., Froehlich J.E., Johnson B.F., Kramer D.M., Jander G., Sharkey T.D. (2016). Rewiring of jasmonate and phytochrome B signalling uncouples plant growth-defense tradeoffs. Nat. Commun..

[97] Guo Q., Yoshida Y., Major I.T., Wang K., Sugimoto K., Kapali G., Havko N.E., Benning C., Howe G.A. (2018). JAZ repressors of metabolic defense promote growth and reproductive fitness in Arabidopsis. Proc. Natl. Acad. Sci. USA.

[98] Munns R., Tester M. (2008). Mechanisms of Salinity Tolerance. Annu. Rev. Plant Biol..

[99] Reddy I.N.B.L., Kim B.K., Yoon I.S., Kim K.H., Kwon T.R. (2017). Salt tolerance in rice: Focus on mechanisms and approaches. Rice Sci..

[100] Moons A., Prinsen E., Bauw G., Van Montagu M. (1997). Antagonistic effects of abscisic acid and jasmonates on salt stress-inducible transcripts in rice roots. Plant Cell.

[101] Kang D.J., Seo Y.J., Lee J.D., Ishii R., Kim K.U., Shin D.H., Park S.K., Jang S.W., Lee I.J. (2005). Jasmonic acid differentially affects growth, ion uptake and abscisic acid concentration in salt-tolerant and salt-sensitive rice cultivars. J. Agron. Crop Sci..

[102] Wu H., Ye H., Yao R., Zhang T., Xiong L. (2015). OsJAZ9 acts as a transcriptional regulator in jasmonate signaling and modulates salt stress tolerance in rice. Plant Sci..

[103] Martínez-Atienza J., Jiang X., Garciadeblas B., Mendoza I., Zhu J.K., Pardo J.M., Quintero F.J. (2007). Conservation of the salt overly sensitive pathway in rice. Plant Physiol..

[104] Kronzucker H.J., Coskun D., Schulze L.M., Wong J.R., Britto D.T. (2013). Sodium as nutrient and toxicant. Plant Soil.

[105] Toda Y., Tanaka M., Ogawa D., Kurata K., Kurotani K., Habu Y., Ando T., Sugimoto K., Mitsuda N., Katoh E. (2013). RICE SALT SENSITIVE3 forms a ternary complex with JAZ and class-C bHLH factors and regulates jasmonate-induced gene expression and root cell elongation. Plant Cell.

[106] Kurotani K., Hayashi K., Hatanaka S., Toda Y., Ogawa D., Ichikawa H., Ishimaru Y., Tashita R., Suzuki T., Ueda M. (2015). Elevated levels of *CYP94* family gene expression alleviate the jasmonate response and enhance salt tolerance in rice. Plant Cell Physiol..

[107] Kurotani K., Yamanaka K., Toda Y., Ogawa D., Tanaka M., Kozawa H., Nakamura H., Hakata M., Ichikawa H., Hattori T. (2015). Stress tolerance profiling of a collection of extant salt-tolerant rice varieties and transgenic plants overexpressing abiotic stress tolerance genes. Plant Cell Physiol..

[108] Hazman M., Sühnel M., Schäfer S., Zumsteg J., Lesot A., Beltran F., Marquis V., Herrgott L., Miesch L., Riemann M. (2019). Characterization of Jasmonoyl-Isoleucine (JA-Ile) hormonal catabolic pathways in rice upon wounding and salt stress. Rice.

[109] Hazman M., Hause B., Eiche E., Nick P., Riemann M. (2015). Increased tolerance to salt stress in OPDA-deficient rice *ALLENE OXIDE CYCLASE* mutants is linked to an increased ROS-scavenging activity. J. Exp. Bot..

[110] Seo J.S., Joo J., Kim M.J., Kim Y.K., Nahm B.H., Song S.I., Cheong J.J., Lee J.S., Kim J.K., Choi Y.D. (2011). OsbHLH148, a basic helix-loop-helix protein, interacts with OsJAZ proteins in a jasmonate signaling pathway leading to drought tolerance in rice. Plant J..

[111] Fu J., Wu H., Ma S., Xiang D., Liu R., Xiong L. (2017). *OsJAZ1* attenuates drought resistance by regulating JA and ABA signaling in rice. Front. Plant Sci..

[112] Dhakarey R., Raorane M.L., Treumann A., Peethambaran P.K., Schendel R.R., Sahi V.P., Hause B., Bunzel M., Henry A., Kohli A. (2017). Physiological and proteomic analysis of the rice mutant *cpm2* suggests a negative regulatory role of jasmonic acid in drought tolerance. Front. Plant Sci..

[113] Hu Y., Jiang L., Wang F., Yu D. (2013). Jasmonate regulates the INDUCER OF CBF EXPRESSION-C-REPEAT BINDING FACTOR/DRE BINDING FACTOR1 cascade and freezing tolerance in Arabidopsis. Plant Cell.

[114] Thomashow M.F. (2010). Molecular basis of plant cold acclimation: Insights gained from studying the CBF cold response pathway. Plant Physiol..

[115] Deng C., Ye H., Fan M., Pu T., Yan J. (2017). The rice transcription factors OsICE confer enhanced cold tolerance in transgenic Arabidopsis. Plant Signal. Behav..

[116] Mao D., Xin Y., Tan Y., Hu X., Bai J., Liu Z.Y., Yu Y., Li L., Peng C., Fan T. (2019). Natural variation in the *HAN1* gene confers chilling tolerance in rice and allowed adaptation to a temperate climate. Proc. Natl. Acad. Sci. USA.

[117] Kobayashi T., Itai R.N., Senoura T., Oikawa T., Ishimaru Y., Ueda M., Nakanishi H., Nishizawa N.K. (2016). Jasmonate signaling is activated in the very early stages of iron deficiency responses in rice roots. Plant Mol. Biol..

[118] Kobayashi T., Nakanishi Itai R., Nishizawa N.K. (2014). Iron deficiency responses in rice roots. Rice.

[119] Singh A.P., Pandey B.K., Deveshwar P., Narnoliya L., Parida S.K., Giri J. (2015). JAZ Repressors: Potential involvement in nutrients deficiency response in rice and Chickpea. Front. Plant Sci..

[120] Wu X., Ding C., Baerson S.R., Lian F., Lin X., Zhang L., Wu C., Hwang S.Y., Zeng R., Song Y. (2019). The roles of jasmonate signalling in nitrogen uptake and allocation in rice *(Oryza sativa* L.). Plant Cell Environ..

[121] Ismail A., Seo M., Takebayashi Y., Kamiya Y., Eiche E., Nick P. (2014). Salt adaptation requires efficient fine-tuning of jasmonate signalling. Protoplasma.

[122] Wasternack C., Strnad M. (2018). Jasmonates: News on occurrence, biosynthesis, metabolism and action of an ancient group of signaling compounds. Int. J. Mol. Sci..

[123] Howe G.A., Major I.T., Koo A.J. (2018). Modularity in jasmonate signaling for multistress resilience. Annu. Rev. Plant Biol..

