# Supplementary material for: Jasmonates—the Master Regulator of Rice Development, Adaptation and Defense
Source: Plants (Basel). 2019 Sep 9;8(9):339. doi: 10.3390/plants8090339 (PMC6784130; doi:10.3390/plants8090339)
Supplement: Supplementary file 1 [file plants-08-00339-s001.pdf]

Table S1. Mutant and transgenic plants in jasmonates biosynthesis and signaling in rice

Gene name	Name of mutant/ transgenic lines	RAP ID	Function	Reference
OsHI-LOX	*oshiloxRNAi*	Os08g0508800	**JA biosynthesis**<br>Increases susceptibility to chewing herbivores, but enhances resistance to a phloem feeder when silenced.	[62]
OsAOS1	*pre/cpm1*<br>*(precocious/ coleoptile morphogenesis 1)*	Os03g0767000	**JA biosynthesis**<br>The *pre/cpm1* plant has long leaves and elongated coleoptiles and mesocotyls, flowers approximately 5 days earlier with the flowers remaining open after flowering, and is partially sterile.	[32,33]
OsAOC	*cpm2/hebiba*<br>*(coleoptile morphogenesis 2)*	Os03g0438100	**JA biosynthesis**<br>The mutation in the *OsAOC* gene produces abnormalities in the spikelet architecture with elongated palea, sterile lemmas, additional bract-like organs, additional anthers and pistils, an early heading time, and elongated coleoptiles and mesocotyls.<br>Increases susceptibility to *Magnaporthe grisea*, the root-knot nematode *Meiloidogyne graminicola*, the parasitic plant *Striga hermonthica*, and the generalist cucumber beetle *Diabrotica balteata*<br>Enhances drought and salt tolerance.	[29,30,60,70,76]
OsOPR7	*og1/ucgl*<br>(*open glume 1 /unclose glume)*	Os08g0459600	**JA biosynthesis**<br>The mutation in *OsOPR7* results in the lemma and palea not being able to fully close during anthesis and malformed, shriveled, and mildewed seeds within open glumes.	[34,35]
OsJAR1	*osjar1*	Os05g0586200	**Catalyzes the conjugation of JA and an amino acid**<br>Loss of function of *OsJAR1* and defects in husk closure and anther dehiscence.	[36]
OsCYP94C2b	*CYP94C2b*_OE	*Os12g0150200*	**Catalyzes the conversion of JA-Ile into 12-OH-JA-Ile and 12-COOH-JA-Ile**<br>Increases salt tolerance and increases internode length and plant height under normal conditions when overexpressed.	[106,107]
OsJAZ1	*eg2*<br>(*extra glume 2*)	Os04g0653000	**Repressor in JA signaling**<br>Increases floret number, culm length, and grain weight; reduces heading time and spikelet fertility; and enhances root growth when overexpressed.<br>Produces extra glume-like structures, alters floral organ numbers and identities, and increases root length when mutated.	[31,45,46]

OsJAZ3	*OsJAZ3*_OE	Os08g0428400	**Repressor in JA signaling**<br>Increased number of organs in florets, aberrant patterns of organ formation, and repetitious organ production in spikelets when overexpressed in the dominant negative form.	[44]
OsJAZ5	*OsJAZ5*_OE	Os04g0395800	**Repressor in JA signaling**<br>Increased floret number, culm length, and grain weight and reduced heading time and spikelet fertility when overexpressed.	[45]
OsJAZ6	*OsJAZ6*_OE	Os03g0402800	**Repressor in JA signaling**<br>Increased floret number, culm length, and grain weight and reduced heading time and spikelet fertility when overexpressed.<br>Increased number of organs in florets, aberrant patterns of organ formation, and repetitious organ production in spikelets when overexpressed in the dominant negative form.	[44,45]
OsJAZ7	*OsJAZ7*_OE	Os07g0615200	**Repressor in JA signaling**<br>Increased floret number, culm length, grain weight, and heading time when overexpressed.<br>Increased number of organs in florets, aberrant patterns of organ formation, and repetitious organ production in spikelets when overexpressed in the dominant negative form.	[44,45]
OsJAZ8	*OsJAZ8*_OE<br>*OsJAZ8ΔC*	Os09g0439200	**Repressor in JA signaling**<br>Increased floret number and culm length; reduced heading time, spikelet fertility, and grain weight when overexpressed.<br>Insensitive to JA treatment and loses JA-induced resistance to *Xanthomonas oryzae* pv *oryzae* when overexpressed in the dominant negative form.	[45,57]
OsJAZ9	*OsJAZ9*_OE<br>*osjaz9*	Os03g0180800	**Repressor in JA signaling**<br>Increased floret number, culm length, and grain weight; reduced heading time and spikelet fertility and increased salt tolerance when overexpressed.<br>Increases susceptibility to salt stress when mutated.	[10,45,102]
OsJAZ10	*OsJAZ10*_OE	Os03g0181100	**Repressor in JA signaling**<br>Increased floret number, culm length, and grain weight; reduced heading time and spikelet fertility when overexpressed.	[45]
OsJAZ11	*OsJAZ11*_OE	Os03g0180900	**Repressor in JA signaling**<br>Increased floret number, culm length, and grain weight; a reduced number of days to flowering and spikelet fertility when overexpressed.	[44,45]

			Increased number of organs in florets, aberrant patterns of organ formation, and repetitious organ production in spikelets when overexpressed in the dominant negative form.	
OsJAZ12	*OsJAZ12*_OE	Os10g0392400	**Repressor in JA signaling**<br>Increased floret number, culm length, and grain weight; reduced number of days to flowering and spikelet fertility when overexpressed.	[45]
OsJAZ13	*OsJAZ13*_OE	Os10g0391400	**Repressor in JA signaling**<br>Increased floret number; reduced number of days to flowering, grain weight, culm length, and spikelet fertility when overexpressed.	[45]
OsCOI1a	*oscoi1aRNAi*	Os01g0853400	**Receptor of JA-Ile**<br>Increased plant height and grain length and increased susceptibility to the rice leaf folder (LF) *Cnaphalocrocis medinalis* and the *Rice black streaked dwarf virus* (RBSDV) when silenced.	[39,63,75–41]
OsCOI1b	*oscoi1b*	Os05g0449500	**Receptor of JA-Ile**<br>Delays leaf senescence and reduces fertility and grain filling rate when mutated.<br>Increases plant height and grain length and increases susceptibility to the *Rice black streaked dwarf virus* (RBSDV) when silenced.	[39,40,75]
OsNINJA1	*OsNINJA1*_OE	Os05g0558800	**Component of a corepressor complex and connects JAZ and NINJA proteins in JA signaling**<br>Increases susceptibility to the bacterial blight disease caused by *Xanthomonas oryzae* pv *oryzae*, and delays leaf senescence when overexpressed.	[47]
OsMYC2	*OsMYC2*_OE<br>*osmyc2RNAi*	Os10g0575000	**Activator of a transcription factor in JA signaling**<br>Regulates senescence-associated genes, inhibits seedling growth, and increases resistance to *Xanthomonas oryzae* pv *oryzae* when overexpressed.<br>Reduces expression of JA-responsive genes when silenced.	[48, 53]
OsRSS3	*rss3 (rice salt sensitive 3)*	Os11g0446000	*RSS3*’s function is required for root cell elongation and the control of root growth under salinity conditions. A loss of function of *RSS3* moderately inhibits cell elongation under normal conditions, but it provokes spontaneous root cell swelling, accompanied by severe root growth inhibition, under saline conditions.	[1056]
